# Bifurcation Control of an Electrostatically-Actuated MEMS Actuator with Time-Delay Feedback

**DOI:** 10.3390/mi7100177

**Published:** 2016-10-01

**Authors:** Lei Li, Qichang Zhang, Wei Wang, Jianxin Han

**Affiliations:** 1 Tianjin Key Laboratory of Nonlinear Dynamics and Chaos Control, Tianjin University, Tianjin 300072, China; lleisnowflake@gmail.com (L.L.); qzhang@tju.edu.cn (Q.Z.); 2 Department of Mechanics, School of Mechanical Engineering, Tianjin University, Tianjin 300072, China; 3 Tianjin Key Laboratory of High Speed Cutting and Precision Machining, Tianjin University of Technology and Education, Tianjin 300222, China; hanjianxin@tju.edu.cn

**Keywords:** MEMS actuators, bifurcations, multiple scales, parametric resonance, time-delay control, monostable vibration

## Abstract

The parametric excitation system consisting of a flexible beam and shuttle mass widely exists in microelectromechanical systems (MEMS), which can exhibit rich nonlinear dynamic behaviors. This article aims to theoretically investigate the nonlinear jumping phenomena and bifurcation conditions of a class of electrostatically-driven MEMS actuators with a time-delay feedback controller. Considering the comb structure consisting of a flexible beam and shuttle mass, the partial differential governing equation is obtained with both the linear and cubic nonlinear parametric excitation. Then, the method of multiple scales is introduced to obtain a slow flow that is analyzed for stability and bifurcation. Results show that time-delay feedback can improve resonance frequency and stability of the system. What is more, through a detailed mathematical analysis, the discriminant of Hopf bifurcation is theoretically derived, and appropriate time-delay feedback force can make the branch from the Hopf bifurcation point stable under any driving voltage value. Meanwhile, through global bifurcation analysis and saddle node bifurcation analysis, theoretical expressions about the system parameter space and maximum amplitude of monostable vibration are deduced. It is found that the disappearance of the global bifurcation point means the emergence of monostable vibration. Finally, detailed numerical results confirm the analytical prediction.

## 1. Introduction

Microelectromechanical systems (MEMS) have been widely applied in gyroscopes [1,2], filter [3,4,5] and so on. Parametric resonance in MEMS was first proposed for amplification of harmonically-excited oscillators [6], and since then parametric excitation has been investigated for increasing sensitivity in scanning probe microscopy [7], mass sensing [8], and tuning [9,10]. With the existence of structure nonlinearity and nonlinear electrostatic forces, they can exhibit rich static and dynamic behaviors [11], such as nonlinear jump phenomena [12] and chaos [13]. Rhoads et al. [14] studied a single degree of freedom parametric excitation equation with both the linear and cubic terms and provided a complete description of the dynamic response. Welte et al. [15] studied parametric excitation in a two degree of freedom MEMS. Design parameters were included in the model by lumping them into non-dimensional parameters, thereby allowing for an easier understanding of their effects and the interaction between the mechanical and electrical forces. A variety of nonlinear dynamic behaviors exist in the nonlinear parametric excitation system. However, only a small part of the dynamic behaviors are desired, which requires us to control the bifurcation behavior of the system. 

To control the dynamic behavior of the system, researchers proposed various parameter optimization methods; for instance, choosing the correct geometry and appropriate voltage. However, it is not enough to improve the performance of the system. To enhance the actuator performance, tip tracking control [16], time-delayed feedback control [17], and pole placement control [18] were introduced. Here, we mainly care about the performance of a time delay feedback controller. Generally, the delayed signal can be displacement, velocity [19], and acceleration [20]. With a properly designed time delay, a delayed feedback controller has been proven to stabilize systems, including atomic force microscopes (AFM) [21] and magneto-elastic beam systems [22]. Shehrin et al. [23] proposed the active control of effective stiffness, damping, and mass of MEMS by applying feedback forces that are proportional to displacement, velocity, and acceleration of its proof mass. Mehta et al. [24] used position feedback to control the effective stiffness of a micro-cantilever to improve the quality factor for biological sensing applications. Morrison et al. [25] investigated the dynamic behaviors of a delayed nonlinear Mathieu equation, and the method of averaging (valid for small ε) was used to obtain a slow flow that was analyzed for stability and bifurcation. Alsaleem et al. [26] presented a study for the stabilization of a MEMS resonator by using a delayed feedback controller. The controller showed a good performance in rejecting disturbances. Warminski [27] analyzed vibrations of a parametrically-excited MEMS device driven by external excitation and time delay inputs. Alsaleem et al. [28] investigated the stability and integrity of parallel-plate MEMS resonators by using a delayed feedback controller. The perturbation method plays an important role in the nonlinear dynamic analysis of MEMS resonators [14]. Kaminski et al. [29,30] studied stochastic nonlinear dynamic behaviors of a MEMS device using the generalized stochastic perturbation technique. 

It can be concluded from the above analysis that nonlinear dynamic behaviors and parameter optimization are both important in the design of parametric excitation MEMS and should be taken into account [31,32,33]. Meanwhile, a time delay feedback controller is gradually applied to the design of the MEMS. However, to the best of our knowledge, there are fewer quantitative studies about a general analysis of parametric excitation comb systems with time delay feedback controllers. Additionally, the parametric excitation system consisting of flexible beam and shuttle mass widely exists in MEMS. Early studies mainly focus on a single degree of freedom, which cannot accurately describe the nonlinear dynamic behaviors. In this paper, a new method is used to solve partial differential equations and the detailed mathematical derivation is proposed to quantitatively make a complete description of the transition mechanism of nonlinear jumping phenomena. It is noteworthy that we are mainly concerned with the nonlinear dynamic behavior of MEMS actuator. Here, the influence of parasitic capacitance is not considered.

The structure of this paper is as follows: in Section 2, the partial differential governing equation with parametric excitation and time-delay feedback is obtained; in Section 3, we apply the method of multiple scales directly to the partial differential equation to produce an approximate solution; in Section 4, we analyze the stability and bifurcation near the origin and the discriminant of Hopf bifurcation is theoretically derived; in Section 5, the global bifurcation and saddle note bifurcation are studied. With a time delay feedback controller, monostable vibration is realized. In Section 6, the numerical simulation is given; and Concluding remarks are given in Section 7. 

## 2. Problem Formulation

**Figure 1:**
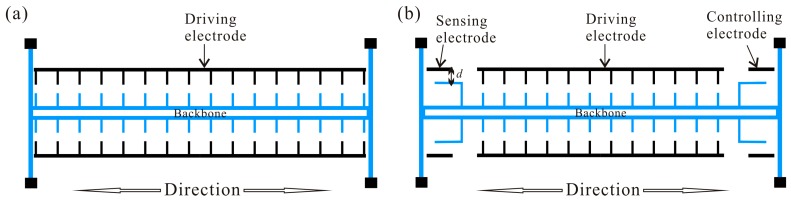
The traditional parametrically-excited MEMS schematic diagram (**a**); and the parametrically-excited multiple electrodes microelectromechanical systems (MEMS) schematic diagram (**b**). (**blue**: movable element; **black**: stationary element).

**Figure 2:**
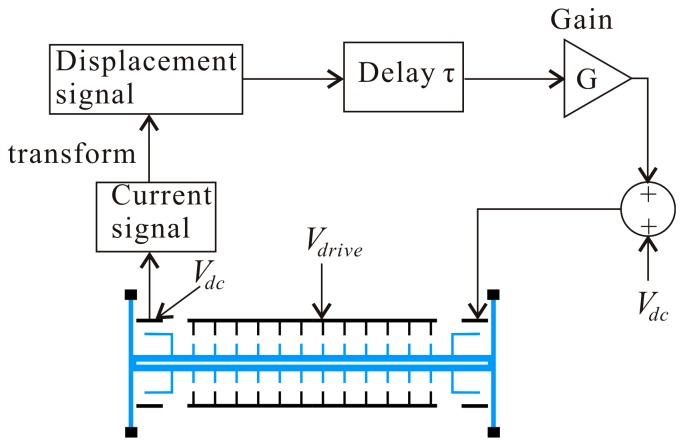
Schematic for the time delayed feedback controller. (**blue**: movable element; **black**: stationary element).

Parametrically-excited MEMS was proposed for use in a number of sensing, actuating, resonator, and filtering applications. A conceptual model of an electrostatic comb-finger actuator is shown in Figure 1a, which has been investigated in many studies [5,14]. Such actuators consist of a shuttle mass, namely the actuator’s backbone, connected to anchors via beam springs, and excited by a pair of non-interdigitated electrostatic comb drives, which are powered by a voltage source. The actuator’s motion is assumed to be described by the movement of the shuttle mass in one direction in the plane. In this paper, multiple electrodes are introduced to optimize the dynamic behavior [34], as shown in Figure 1b. Here, the electrostatic comb-finger actuator is driven by a sensing electrode, a driving electrode, and a controlling electrode. Morrison et al. [25] proved that time delay feedback force can control stability and bifurcation in parametrically-excited nonlinear differential equations. In order to improve the global dynamic behavior of the system, the time delay feedback is introduced as shown in Figure 2, where τ is the time delay and *G* is the amplitude of the displacement feedback controller with unit V/m.

The electrostatic attractive force is derived from the very well-known equation:

(1) $$F=-\frac{\partial E}{\partial x}$$

where *E* is the electric energy stored in the capacitor, i.e., *E = CV^2^*/2. Here, *C* represents capacitance.

The electrostatic force arising from the driving electrode can be modeled for small displacements [14]:

*F*_drive_ = (*r*_1_*w* + *r*_3_*w*^3^)*V*^2^(1 + cosΩ*t*) (2)

where *r*_1_ and *r*_3_ are electrostatic coefficients, which can provide harmonic excitation to the device. Here, *w* is displacement of shuttle mass and *V* is driving voltage.

Then, the electrostatic forces arising from the sensing electrode and controlling electrode can be derived using Equation (1) as [12]:

(3) $$F_{\mathrm{sense}}=\frac{\varepsilon_{0}h}{d}V_{dc}^{2}$$

(4) $$F_{\mathrm{control}}=-\frac{\varepsilon_{0}h}{d}{\left(V_{dc}+Gw(t-\tau )\right)}^{2}$$

where *d* is the gap width, *h* is thickness of the microbeam and ε_0_ is the dielectric constant of the gap medium. Here, we consider *V_dc_* >> *Gw*(*t* − *τ*). Then *F*_control_ = $-\frac{\varepsilon_{0}h}{d}$ ($V_{dc}^{2}$ + 2*V_dc_Gw*(*t* − *τ*)) is obtained.

**Figure 3:**
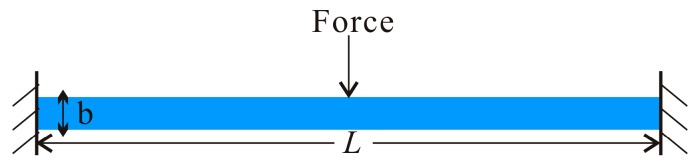
The equivalent mechanical model schematic diagram. (**blue**: movable element; **black**: stationary element).

The electrostatic comb-finger actuator can be described by two clamped-clamped beams and a shuttle mass. An equivalent mechanical model is shown in Figure 3. In this paper, we consider a clamped-clamped Euler microbeam actuated by a concentrated load and subject to a viscous damping *c* per unit length. By using Hamilton’s principle, the equation of motion that governs the transverse deflection *w*(*x*,*t*) is written as [35]:

(5) $$\rho A\ddot{w}+EIw^{\mathrm{iv}}+c\dot{w}-(\frac{EA}{2L}\int_{0}^{L}{w^{\prime }}^{2}dx)w^{\prime \prime }=\frac{1}{2}(-M\ddot{w}-F_{\mathrm{drive}}-F_{\mathrm{control}}-F_{\mathrm{sense}})\delta (x-\frac{1}{2}L)$$

with the boundary conditions:

*w*(0,*t*) = *w’*(0,*t*) = *w’*(*L*,*t*) = *w*(*L*,*t*) (6)

where $\dot{w}=\partial w/\partial t$ and $w^{\prime }=\partial w/\partial x$, *x* is the position along the plate length, *A* and *I* are the area and moment of inertia of the microbeam, *t* is time, *E* is Young’s modulus, ρ is the material density, *L* is the microbeam length, *b* is the microbeam width, *M* is mass of shuttle, and δ represents impulse function.

For convenience, we introduce the non-dimensional variables:

$$w_{n}=\frac{w}{x_{0}},\;t_{n}=\frac{t}{\sqrt{\frac{\rho AL^{4}}{EI}}},\;x_{n}=\frac{x}{L}$$

where *x_0_* is a characteristic length of the system. *x*_0_ = 2 μm is taken.

Substituting the non-dimensional variables into Equations (5) and (6), yields the following non-dimensional equation of motion:

(7) $$\begin{array}{l} {\ddot{w}}_{n}+w_{n}^{\mathrm{iv}}+c_{n}{\dot{w}}_{n}-\frac{Ax_{0}^{2}}{2I}(\int_{0}^{1}{w_{n}^{\prime }}^{2}dx_{n})w_{n}^{\prime \prime }\\ =-[M_{n}{\ddot{w}}_{n}+(r_{1n}w_{n}+r_{3n}{w_{n}}^{3})V^{2}(1+\cos\Omega_{n}t_{n})-\beta_{n}w_{n}(t_{n}-\tau_{n})]\delta (x_{n}-\frac{1}{2}) \end{array}$$

with boundary conditions:

(8) $$w_{n}(0,t_{n})=w_{n}^{\prime }(0,t_{n})=w_{n}^{\prime }(1,t_{n})=w_{n}(1,t_{n})=0$$

where, $c_{n}=cL^{2}/\sqrt{EI\rho A}$, $M_{n}=M/2\rho AL$, $r_{1n}=r_{1}L^{3}/2EI$, $r_{3n}=r_{3}L^{3}x_{0}^{2}/2EI$, $\beta_{n}=2\beta L^{3}/EI$, $\beta =\varepsilon_{0}hV_{dc}G/2d$. Here, β represents the time delay feedback gain of the system.

## 3. Perturbation Analysis

In this section, the method of multiple scales [36] are directly used to investigate the response of the MEMS actuator with small vibration amplitude around an equilibrium position. To indicate the significance of each term in the equation of motion, ε is introduced as a small non-dimensional bookkeeping parameter. Considering that the inertia force of the flexible beam is much smaller than that of the shuttle mass, ${\ddot{w}}_{n}=O(\varepsilon )$ is given. Then, scaling the damping, nonlinear electrostatic force, periodic excitation, and time delay feedback force, we obtain:

(9) $$\begin{array}{l} \varepsilon {\ddot{w}}_{n}+w_{n}^{iv}+\varepsilon c_{n}{\dot{w}}_{n}-\varepsilon \alpha (\int_{0}^{1}{w_{n}^{\prime }}^{2}dx_{n})w_{n}^{\prime \prime }\\ =-\delta (x_{n}-\frac{1}{2})[M_{n}{\ddot{w}}_{n}+r_{1n}w_{n}V^{2}+\varepsilon r_{1n}w_{n}V^{2}\cos\Omega_{n}t_{n}\\ +\varepsilon r_{3n}w_{n}^{3}V^{2}(1+\cos\Omega_{n}t_{n})-\varepsilon \beta_{n}w_{n}(t_{n}-\tau_{n})] \end{array}$$

with the boundary conditions:

$$w_{n}(0,t_{n})=w_{n}^{\prime }(0,t_{n})=w_{n}^{\prime }(1,t_{n})=w_{n}(1,t_{n})=0$$

where $\alpha =Ax_{0}^{2}/2I$.

In order to investigate the primary parametric resonance when the driving frequency is close to two times of the natural frequency, a detuning parameter σ is introduced and defined by:

Ω*_n_* = 2ω + εσ (10)

where ω is the natural frequency of the system.

We seek the approximate solution of Equation (9) in the form:

*w_n_* = *w*_0_(*x_n_*,*T*_0_,*T*_1_) + ε*w*_1_(*x_n_*,*T*_0_,*T*_1_) (11)

where *T*_0_ = *t* and *T*_1_ = ε*t*.

Substituting Equations (11) and (10) into Equation (9) and equating coefficients of like powers of ε^0^ and ε^1^, yield

ε^0^:

(12) $$w_{0}^{iv}=-\delta (x_{n}-\frac{1}{2})(M_{n}\frac{\partial^{2}w_{0}}{\partial T_{0}^{2}}+r_{1n}w_{0}V^{2})$$

(13) $$w_{0}(0,t_{n})=w_{0}^{\prime }(0,t_{n})=w_{0}(1,t_{n})=w_{0}^{\prime }(1,t_{n})=0$$

ε^1^:

(14) $$\begin{array}{l} w_{1}^{iv}+\frac{\partial^{2}w_{0}}{\partial T_{0}^{2}}+c_{n}\frac{\partial w_{0}}{\partial T_{0}}-\alpha (\int_{0}^{1}{w_{0}^{\prime }}^{2}dx_{n})w_{0}^{\prime \prime }\\ =-\delta (x_{n}-\frac{1}{2})[2M_{n}\frac{\partial^{2}w_{0}}{\partial T_{0}\partial T_{1}}+M_{n}\frac{\partial^{2}w_{1}}{\partial T_{0}^{2}}+r_{1n}w_{1}V^{2}+r_{1n}w_{0}V^{2}\cos\Omega_{n}t_{n}\\ +r_{3n}w_{0}^{3}V^{2}(1+\cos\Omega_{n}t_{n})-\beta_{n}w_{0}(t_{n}-\tau_{n})] \end{array}$$

(15) $$w_{1}(0,t_{n})=w_{1}^{\prime }w_{1}(1,t_{n})=w_{1}^{\prime }(1,t_{n})=0$$

Considering a single mode vibration, the general solution of Equation (12) can be written as:

(16) $$w_{0}=\varphi_{0}(x_{n})A(T_{1})e^{i\omega T_{0}}+\mathrm{cc}$$

where cc indicates the complex conjugate of the preceding terms.

Substituting Equation (16) into Equations (12) and (13), yields:

(17) $$w_{0}=\{\begin{array}{cc} \left(-16{x_{n}}^{3}+12{x_{n}}^{2})(A(T_{1})e^{i\omega T_{0}}+\mathrm{cc}\right) & x_{n}\in [0,\;1/2]\\ \left(16{x_{n}}^{3}-36{x_{n}}^{2}+24x_{n}-4)(A(T_{1})e^{i\omega T_{0}}+\mathrm{cc}\right) & x_{n}\in (1/2,\;1] \end{array}$$

where ω can be defined by Equation (12). We obtain $\omega =\sqrt{\left(192+r_{1n}V^{2}\right)/M_{n}}$.

The general solution of Equation (14) can be written as:

(18) $$w_{1}=\varphi_{1}(x_{n},T_{1})u_{1}(T_{0})$$

Substituting Equation (18) into Equation (14), multiplying by $\varphi_{0}$, and integrating the outcome from *x* = 0 to 1, we obtain [36,37]:

(19) $$\begin{array}{l} ({\ddot{u}}_{1}+\omega^{2}u_{1})M_{n}\\ ={-[i2\omega M_{n}\frac{dw_{0}}{dT_{1}}+r_{1n}w_{0}V^{2}\cos\Omega_{n}t_{n}\;+r_{3n}{w_{0}}^{3}V^{2}(1+\cos\Omega_{n}t_{n})-\beta_{n}w_{0}(t_{n}-\tau_{n})]|}_{x_{n}=\frac{1}{2}}\\ +\int_{0}^{1}[\omega^{2}w_{0}-c_{n}i\omega w_{0}+\alpha (\int_{0}^{1}{w_{0}^{\prime }}^{2}dx_{n}){w^{\prime \prime }}_{0}]\varphi_{0}dx_{n} \end{array}$$

Substituting Equations (10) and (17) into Equation (19) and eliminating the secular term, we obtain:

(20) $$\begin{array}{l} -\frac{13}{35}\omega^{2}A+\frac{13}{35}c_{n}i\omega A+3A{\left|A\right|}^{2}\alpha \frac{576}{25}+i2\omega M_{n}\frac{dA}{dT_{1}}+3r_{3n}A{\left|A\right|}^{2}V^{2}+\frac{1}{2}r_{1n}\bar{A}V^{2}e^{i\sigma T_{1}}\\ +\frac{3}{2}r_{3n}\bar{A}{\left|A\right|}^{2}V^{2}e^{i\sigma T_{1}}+\frac{1}{2}r_{3n}A^{3}V^{2}e^{-i\sigma T_{1}}-\beta_{n}Ae^{-i{\omega \tau }_{n}}=0 \end{array}$$

where the overbar indicates the complex conjugate and |A| represents modulus of *A*.

At this point, it is convenient to express *A* in the polar form:

(21) $$A(T_{1})=\frac{1}{2}a(T_{1})e^{i\theta (T_{1})}$$

where *a* is the amplitude.

Substituting Equation (21) into Equation (20), and separating the imaginary and real parts, yields:

(22) $$a^{\prime }=\frac{1}{8}a[-8\xi +(2\lambda_{1}+a^{2}\lambda_{3})\sin2\psi -4g\sin{\omega \tau }_{n}]$$

(23) $$\psi \prime \;=\frac{1}{8}[3a^{2}k_{3}+4k_{1}-4\sigma -4g\cos{\omega \tau }_{n}+(2\lambda_{1}+2a^{2}\lambda_{3})\cos2\psi ]$$

where ψ = θ − σ/2 is the phase of oscillator’s response, and several variables are defined as:

$k_{3}=\frac{r_{3n}V^{2}}{\omega M_{n}}+\frac{576}{25\omega M_{n}}\alpha$, $\;k_{1}=-\frac{13\omega }{35M_{n}}$, $\;g=\frac{\beta_{n}}{\omega M_{n}}\;$, $\lambda_{3}=\frac{r_{3n}V^{2}}{\omega M_{n}}$, $\lambda_{1}=\frac{r_{1n}V^{2}}{\omega M_{n}}$, $\xi =\frac{13c_{n}}{70M_{n}}$.

The steady-state response can be obtained by imposing the condition *a*’ = ψ ′ = 0. The first-order approximate solution is obtained:

(24) $$w_{0}=\{\begin{array}{cc} \left(-16{x_{n}}^{3}+12{x_{n}}^{2})a\cos(\Omega t/2+\psi )+O(\varepsilon \right) & x_{n}\in [0,\;1/2]\\ \left(16{x_{n}}^{3}-36{x_{n}}^{2}+24x_{n}-4)a\cos(\Omega t/2+\psi )+O(\varepsilon \right) & x_{n}\in (1/2,\;1] \end{array}$$

## 4. Bifurcation Control near the Origin

For determining the steady states of this system, it turns out to be advantageous to introduce the new unknown variables. We obtain an alternate form of the equation by transforming from polar coordinates *a* and ψ to rectangular coordinates *u* and *v*, where:

(25) u=cosψ, v=asinψ

It is noted that *u* represents the displacement signal and *v* represents the velocity signal when *t* = 0.

Substituting Equation (25) into Equations (22) and (23), results in the form:

(26) $$u^{\prime }=(-\xi -\frac{g}{2}\sin{\omega \tau }_{n})u+(\frac{\lambda_{1}}{4}-\frac{k_{1}}{2}+\frac{\sigma }{2}+\frac{g}{2}\cos{\omega \tau }_{n})v-\frac{3}{8}k_{3}u^{2}v+(-\frac{3}{8}k_{3}+\frac{\lambda_{3}}{4})v^{3}$$

(27) $$v^{\prime }=(\frac{\lambda_{1}}{4}+\frac{k_{1}}{2}-\frac{\sigma }{2}-\frac{g}{2}\cos{\omega \tau }_{n})u+(-\xi -\frac{g}{2}\sin{\omega \tau }_{n})v+\frac{3}{8}k_{3}uv^{2}+(\frac{3}{8}k_{3}+\frac{\lambda_{3}}{4})u^{3}$$

From the Equations (26) and (27), the Jacobian matrix is obtained:

(28) $$J=\frac{1}{8}\left[\begin{array}{cc} a_{11} & a_{12}\\ a_{21} & a_{22} \end{array}\right]$$

where:

$$\begin{array}{l} a_{11}=-8\xi -4g\sin\omega \tau_{n}\\ a_{12}=2\lambda_{1}-4k_{1}+4\sigma +4g\cos\omega \tau_{n}\\ a_{21}=2\lambda_{1}+4k_{1}-4\sigma -4g\cos\omega \tau_{n}\\ a_{22}=-8\xi -4g\sin\omega \tau_{n} \end{array}$$

### 4.1. Stability of the Origin

The trace and determinant of the Jacobian matrix evaluated at an equilibrium point contain the local stability information. From Equations (22) and (23), $8\xi +4g\sin{\omega \tau }_{n}$ represents the effective damping of the system. When it is negative, all of the solutions are not stable. Here, $8\xi +4g\sin{\omega \tau }_{n}>0$ is considered. For stability of the origin, a critical point generically occurs when Det(*J*) = 0. From Equation (28), the Jacobian matrix value of the origin is obtained:

(29) $$\begin{array}{l} \mathrm{Det}(J)={\left(8\xi +4g\sin\omega \tau_{n}\right)}^{2}-(2\lambda_{1}-4k_{1}+4\sigma +4g\cos\omega \tau_{n})(2\lambda_{1}+4k_{1}-4\sigma -4g\cos\omega \tau_{n})\\ \;={\left(8\xi +4g\sin\omega \tau_{n}\right)}^{2}-4{\lambda_{1}}^{2}+{\left(4k_{1}-4\sigma -4g\cos\omega \tau_{n}\right)}^{2} \end{array}$$

Through Equation (29), it is found that the stability of the origin varies with *g* and τ*_n_*. Here, according to the bifurcation behaviors of the origin, two different situations are divided. Case one: when ${\left(8\xi +4g\sin{\omega \tau }_{n}\right)}^{2}-4{\lambda_{1}}^{2}\geq 0$, the origin is always stable. Case two: when ${\left(8\xi +4g\sin{\omega \tau }_{n}\right)}^{2}-4{\lambda_{1}}^{2}<0$, there exists resonance in the system. When Det(*J*) = 0, the bifurcation points can be obtained:

(30) $$\sigma_{1}=k_{1}-g\cos{\omega \tau }_{n}-\frac{1}{4}\sqrt{4{\lambda_{1}}^{2}-{\left(8\xi +4g\sin{\omega \tau }_{n}\right)}^{2}}$$

(31) $$\sigma_{2}=k_{1}-g\cos{\omega \tau }_{n}+\frac{1}{4}\sqrt{4{\lambda_{1}}^{2}-{\left(8\xi +4g\sin{\omega \tau }_{n}\right)}^{2}}$$

The origin is unstable from σ_1_ to σ_2_, which means resonance occurs. Then, the size of the unstable region is obtained:

(32) $$\sigma_{2}-\sigma_{1}=\frac{1}{2}\sqrt{4\lambda_{1}^{2}-{\left(8\xi +4g\sin{\omega \tau }_{n}\right)}^{2}}$$

From Equations (30)–(32), it is found that a negative feedback gain can increase the resonance frequency and widens the resonance band when ωτ*_n_* < π/2. However, the maximum of the resonance band is λ_1_, which is decided by the linear electrostatic force.

In this paper, the model is improved from the traditional one. Part of the system parameters are defined as stated in Table 1 [5].

**Figure 4:**
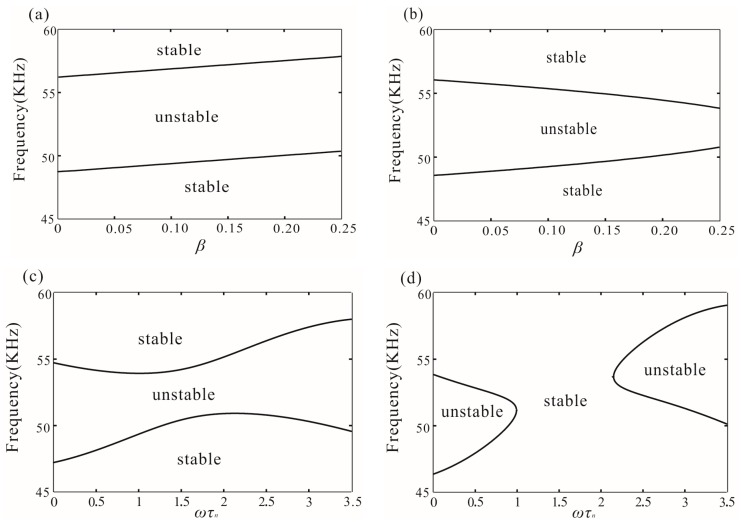
The stability of the origin corresponding to (**a**) the case of ωτ*_n_* = π; (**b**) the case of ωτ*_n_* = π/2; (**c**) the case of β = 0.225; and (**d**) the case of β = 0.36.

Figure 4 shows the stability of origin in the case of *V* = 30 V. It is found that the positive feedback gain can increase the resonance frequency when ωτ_*n*_ = π and decrease the frequency band when ωτ_*n*_ = π/2. Through choosing the right time delay, we can control the resonance frequency and frequency band individually. Figure 4c,d study the resonance frequency and frequency band variance with β = 0.225 and β = 0.36. When feedback gain is more than the critical value λ_1_/2 − 2ξ, the saddle-node bifurcation occurs as shown in Figure 4d. In practical engineering, we can choose the right time delay and gain to meet the engineering demand. 

### 4.2. Stability of the Untrivial Solution

Stability of the origin was studied in the previous section. Through Equations (26) and (27), it is found that Hopf bifurcation will occur at σ_1_ and σ_2_, which can lead to an untrivial solution and change the stability of the origin. Then, stability of the untrivial solution is investigated. The supercritical Hopf bifurcation of σ_1_ and σ_2_ can lead to stable branches between two critical points. On the contrary, subcritical Hopf bifurcation of σ_1_ and σ_2_ can lead to unstable branches. In this section, we study Hopf bifurcation of critical points to determine the stability of periodic vibration. A series of calculations is made in Appendix A, and the discriminants of Hopf bifurcation points are obtained:

(33) $$\Delta_{1}=-3k_{3}\sqrt{4\lambda_{1}^{2}-{\left(8\xi +4g\sin{\omega \tau }_{n}\right)}^{2}}+4\lambda_{1}\lambda_{3}-\frac{\lambda_{3}}{2\lambda_{1}}{\left(8\xi +4g\sin{\omega \tau }_{n}\right)}^{2}$$

(34) $$\Delta_{2}=3k_{3}\sqrt{4\lambda_{1}^{2}-{\left(8\xi +4g\sin{\omega \tau }_{n}\right)}^{2}}+4\lambda_{1}\lambda_{3}-\frac{\lambda_{3}}{2\lambda_{1}}{\left(8\xi +4g\sin{\omega \tau }_{n}\right)}^{2}$$

where $\Delta_{1}$ stands for the discriminant of σ_1_, and $\Delta_{2}$ stands for the discriminant of σ_2_.

The case $\Delta_{1}$ < 0 and $\Delta_{2}$ < 0 results in the supercritical Hopf bifurcation of σ_1_ and σ_2_. Likewise, the case $\Delta_{1}$ > 0 and $\Delta_{2}$ > 0 results in the subcritical Hopf bifurcation of σ_1_ and σ_2_. However, due to the nature of this equation of motion, two mixed cases also exist, namely, $\Delta_{1}$ > 0 and $\Delta_{2}$ < 0, or $\Delta_{1}$ < 0 and $\Delta_{2}$ > 0, which correspond to the two branches from the critical point bending toward each other. When $\Delta_{1}$ = 0 or $\Delta_{2}$ = 0, the type of bifurcation cannot be captured without the inclusion of high-order nonlinear terms, which is not considered here. 

**Figure 5:**
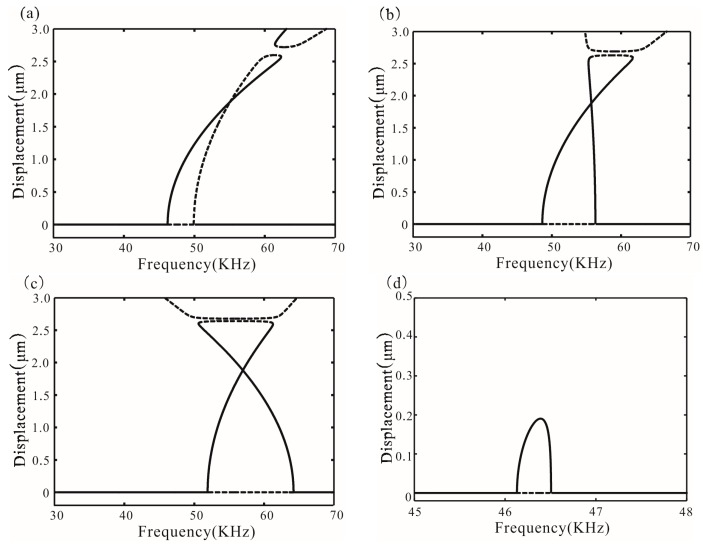
Bifurcation diagram for *V* = 20, 30, and 40 V corresponding to (**a**–**c**) without feedback force (**d**) shows the bifurcation diagram for *V* = 20 V in the case of β = 0.354 and ωτ*_n_* = π/4. Solid lines represent stable amplitude; dotted lines represent unstable amplitude.

Figure 5 shows different types of bifurcation diagrams. The stability of solution can be obtained by Equation (28), as mentioned in [14]. Meanwhile, the values of the discriminant are obtained:

$$\left\{ \begin{array}{l} \Delta_{1}(20)<0\\ \Delta_{2}(20)>0 \end{array}\right.,\left\{ \begin{array}{l} \Delta_{1}(30)<0\\ \Delta_{2}(30)<0 \end{array}\right.,\left\{ \begin{array}{l} \Delta_{1}(40)<0\\ \Delta_{2}(40)<0 \end{array}\right.$$

Among them, $\Delta_{1}$ < 0, supercritical Hopf bifurcation occurs at σ_1_. Similarly, with $\Delta_{2}$(20) > 0, subcritical Hopf bifurcation occurs at σ_2_. With $\Delta_{2}$(30) < 0 and $\Delta_{2}$(40) < 0, supercritical Hopf bifurcation occurs at σ_2_, which coincides with the numerical results of Equations (22) and (23). From *V* = 20 V to *V* = 40 V, the resonance frequency increases. Additionally, feedback force can also change the resonance frequency and transform the types of bifurcation points, as shown in Figure 5d. $\Delta_{2}$(20) < 0 is obtained in the case of β = 0.354 and ωτ*_n_* = π/4. Subcritical Hopf bifurcation of the point σ_2_ is transformed to supercritical Hopf bifurcation. 

**Figure 6:**
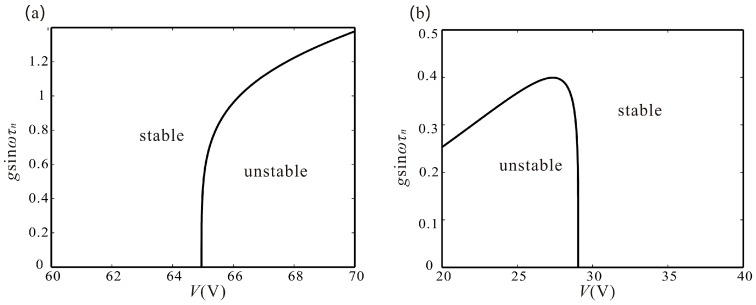
Time-delay feedback control on Hopf bifurcation. (**a**) shows the supercritical Hopf bifurcation area and subcritical Hopf bifurcation area of point σ_1_; and (**b**) shows the supercritical Hopf bifurcation area and subcritical Hopf bifurcation area of point σ_2_.

Through Equations (33) and (34), we can obtain that supercritical Hopf bifurcation always occurs at σ_1_ and σ_2_ when $9k_{3}^{2}-4\lambda_{3}^{2}<0$. However, when $9k_{3}^{2}-4\lambda_{3}^{2}>0$, subcritical Hopf bifurcation may occur at σ_1_ in case of *k*_3_ < 0 and occur at σ_2_ in the case of *k*_3_ > 0. Meanwhile, when $g\sin\omega \tau_{n}$ is close to $\lambda_{1}/2-2\xi$, $\Delta_{1}$ and $\Delta_{2}$ are negative. Thus, with an appropriate feedback force, supercritical Hopf bifurcation always occurs under any driving voltage. The detailed derivation process is seen in Appendix B. Figure 6 shows time delay feedback control on Hopf bifurcation. As shown in Figure 6a, with a voltage value less than 64.96 V, the branch from σ_1_ is always stable. However, the branch becomes unstable with the increase of the voltage. On the contrary, with the voltage value is greater than 29.04 V, the branch from σ_2_ is always stable. However, the branch becomes unstable with the decrease of the voltage, as shown in Figure 6b. With the introduction of the time-delay feedback force, the unstable branches become stable. The curves shown in Figure 6 represent the minimum value of $g\sin\omega \tau_{n}$ to make the branches stable. 

## 5. Monostable Vibration

### 5.1. Global Bifurcation

The monostable vibration, which can eliminate jump phenomena between multiple stable solutions and improve system stability, has been widely applied in engineering, such as resonators. In Section 4, there is one or more periodic solutions under the single frequency. To obtain the parameter space of the monostable vibration, global dynamics analysis is introduced. This is almost unaffected by damping, since it is small, and zero damping is assumed to simplify the analysis.

**Figure 7:**
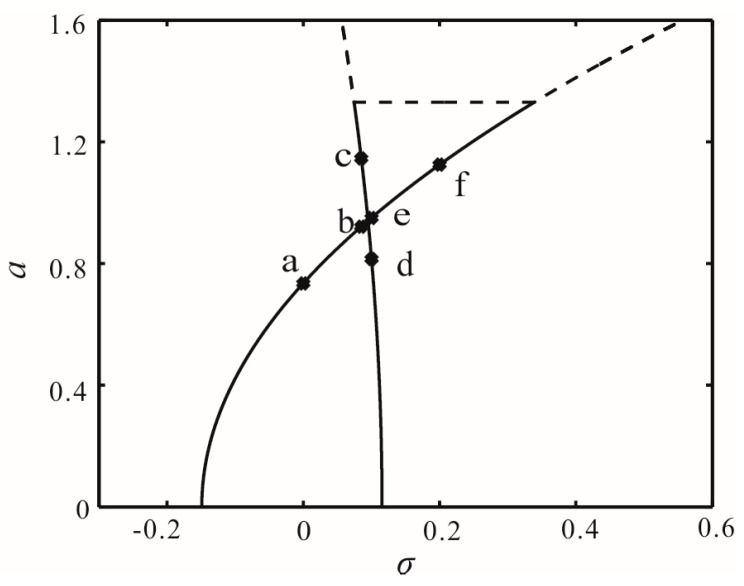
Bifurcation diagram of no damping with *V* = 30 V, **a** is the periodic solution for σ = 0; **b**, **c** are the periodic solutions for σ = 0.085; **d**, **e** are the periodic solutions for σ = 0.1; and **f** is the periodic solution for σ = 0.2.

First, we consider the case of *V* = 30 V without time-delay feedback force. The bifurcation diagram of the no-damping system is shown in Figure 7. Here, four frequency points 0, 0.085, 1, and 2 are taken to analyze the global dynamics. There exists one periodic solution at 0, 2 and two periodic solutions at 0.085, 1, as shown in Figure 7. 

**Figure 8:**
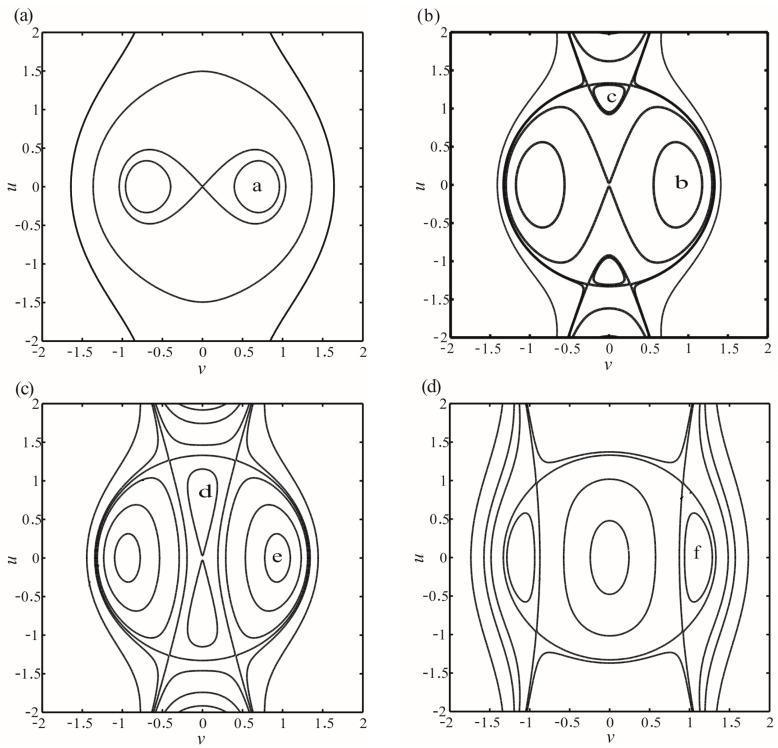
Representative phase planes corresponding to each of the frequency response regimes at *V* = 30 V. **a**, **b**, **c**, **d**, **e**, and **f** correspond to those in Figure 7. (**a**) describes an unstable origin and a center when σ = 0; (**b**) describes an unstable origin and two centers when σ = 0.085; (**c**) describes an unstable origin and two centers when σ = 0.1; and (**d**) describes two centers when σ = 0.2.

Representative phase planes corresponding to each of the frequency response points are delineated in Figure 8. Figure 8a describes an unstable origin and a center, which corresponds to a in Figure 7. Both Figure 8b and Figure 8c depict unstable origins and two centers. It is worth noting that different initial disturbance can lead to different movement tracks. The system easily arrives at b, e by velocity disturbance. Likewise, the system easily arrives at c, d by displacement disturbance. When σ = 0.2, the origin becomes a center, as shown in Figure 8d. From Figure 8b,c, the global bifurcation occurs at the intersection, as shown in Figure 7. Meanwhile, the phenomena of multistability exist in the system.

The above analysis results are based on an undamped system, the existence of the damping brings the center into focus and shifts each of their locations at the same time. 

### 5.2. Saddle Node Bifurcation

Through Equation (22), when the amplitude is in a certain range, there is no periodic vibration, as shown in Figure 5. The existence of periodic vibration requires:

(35) $$-(4g\sin\omega \tau +8\xi )<(2\lambda_{1}+a^{2}\lambda_{3})<4g\sin\omega \tau +8\xi$$

**Figure 9:**
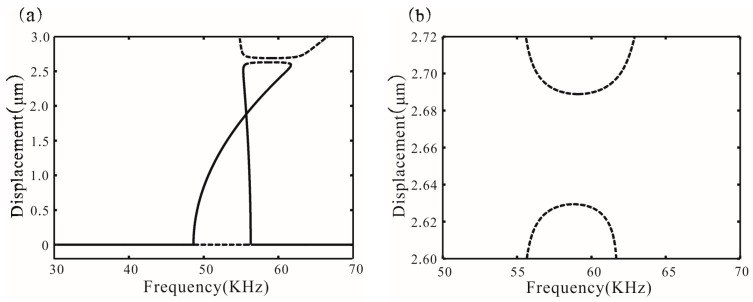
Saddle node bifurcation diagram in the case of *V* = 30 V and β = 0, (**b**) is the local amplification figure of (**a**). Solid lines represent stable amplitude, and dashed lines represent unstable amplitude.

When equal, saddle node bifurcation occurs, as shown in Figure 9. From Equation (23), the perturbation frequency of the node bifurcation point is obtained:

(36) $$\sigma =\frac{3}{4}a^{2}k_{3}+k_{1}-g\cos\omega \tau$$

where:

(37) $$a^{2}=\frac{4g\sin\omega \tau_{n}+8\xi -2\lambda_{1}}{\lambda_{3}}$$

or:

(38) $$a^{2}=\frac{-8\xi -4g\sin\omega \tau_{n}-2\lambda_{1}}{\lambda_{3}}$$

The branch from the bifurcation point σ is unstable, which can be proved by Equations (22) and (23).

### 5.3. Monostable Parameter Vibration

Now, time-delay feedback force is introduced to eliminate the global bifurcation point and make the system translate to a monostable one from a bistable one. When the saddle node bifurcation point and global bifurcation point coincide, the bistable phenomenon disappears, and there is only one stable periodic solution under the single frequency. 

From Equation (23), we obtain:

(39) $$\sigma =\frac{1}{4}[3a^{2}k_{3}+4k_{1}-4g\cos\omega \tau_{n}+(2\lambda_{1}+2a^{2}\lambda_{3})\cos2\psi ]$$

It is found that there is only one amplitude corresponding to one frequency when global bifurcation occurs, which becomes true when 2λ_1_ + 2*a*^2^λ_3_ = 0. In other words, $\sqrt{-\lambda_{1}/\lambda_{3}}$ is the maximum amplitude under monostable parameter vibration, which is only related to the structure parameter. 

When the saddle node bifurcation point and global bifurcation point coincide, the following expression is obtained with Equations (36) and (39):

(40) $$a^{2}=\frac{4g\sin\omega \tau_{n}+8\xi -2\lambda_{1}}{\lambda_{3}}=\frac{-\lambda_{1}}{\lambda_{3}}$$

From Equation (40), it is noted that the maximum amplitude of the system is less than $\sqrt{-\lambda_{1}/\lambda_{3}}$ in the case of $\lambda_{1}/2-2\xi >g\sin\omega \tau_{n}>\lambda_{1}/4-2\xi$. Thus, there is only one periodic solution under this parameter space. 

**Figure 10:**
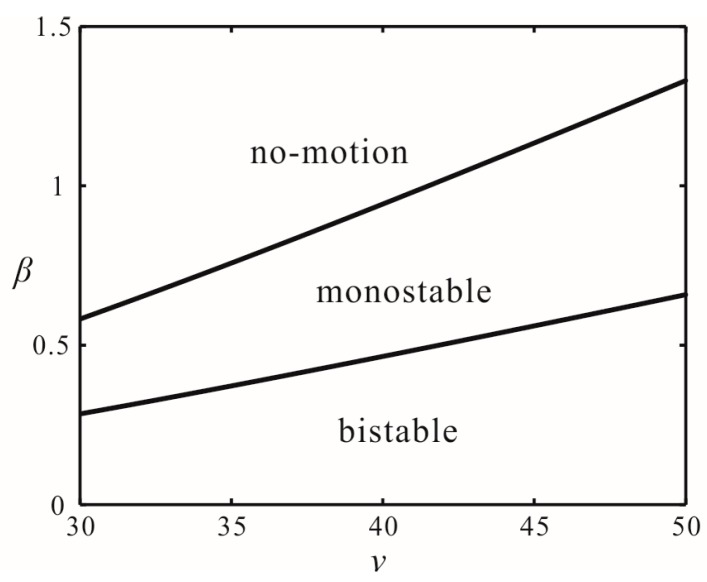
The motion state of the system in the different voltage and gain for ωτ*_n_* = π/2.

**Figure 11:**
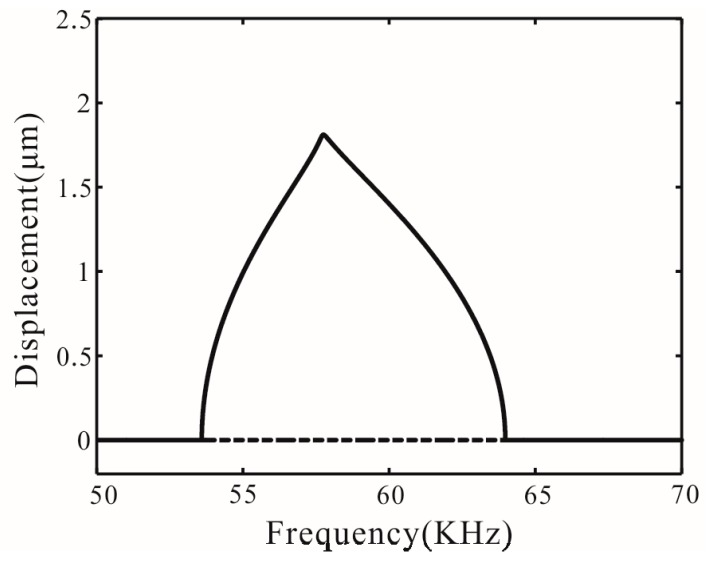
Bifurcation diagram for *V* = 40 V, β = 0.55, and ωτ*_n_* = π/2.

Reasonable design of time-delay feedback in engineering practice can make the system reach a monostable motion state, as shown in Figure 10. This shows the motion state of the system in the different voltage and gain for ωτ*_n_* = π/2. Figure 11 shows the bifurcation diagram of the monostable vibration with *V* = 40 V and β = 0.55.

**Figure 12:**
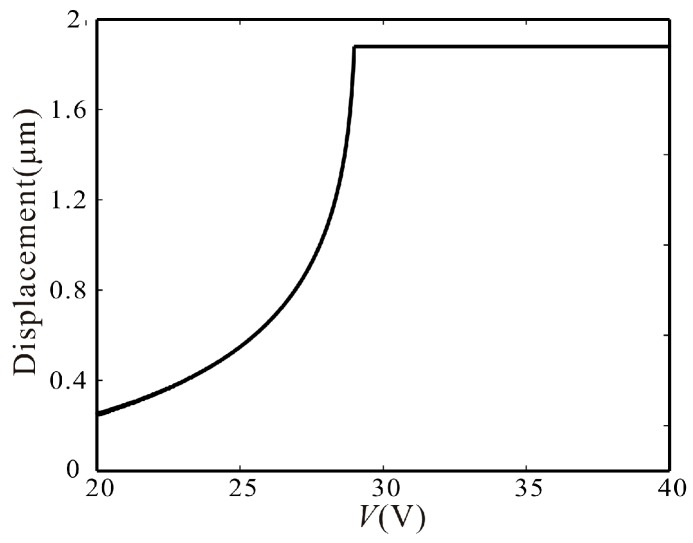
The relationship diagram between the maximum amplitude of the monostable vibration and the voltage.

From Equation (40), although delay feedback force can eliminate the global bifurcation point and make the system translate to a monostable one from a bistable one, it cannot change the maximum amplitude of vibration, which is only related to the structure parameters of the comb. Additionally, in the Section 4, when the voltage is too large or too small, the branches from σ_1_ and σ_2_ become unstable. A large enough force feedback is introduced to make them stable and reduce the maximum amplitude at the same time. In the Figure 12, the relationship between the maximum amplitude of the monostable vibration and the voltage is given. It shows that the maximum amplitude is constant when the voltage is greater than 28.96 V. On the contrary, the maximum amplitude decreases with the decrease of voltage when the voltage is less than 28.96 V. 

## 6. Numerical Simulation

**Figure 13:**
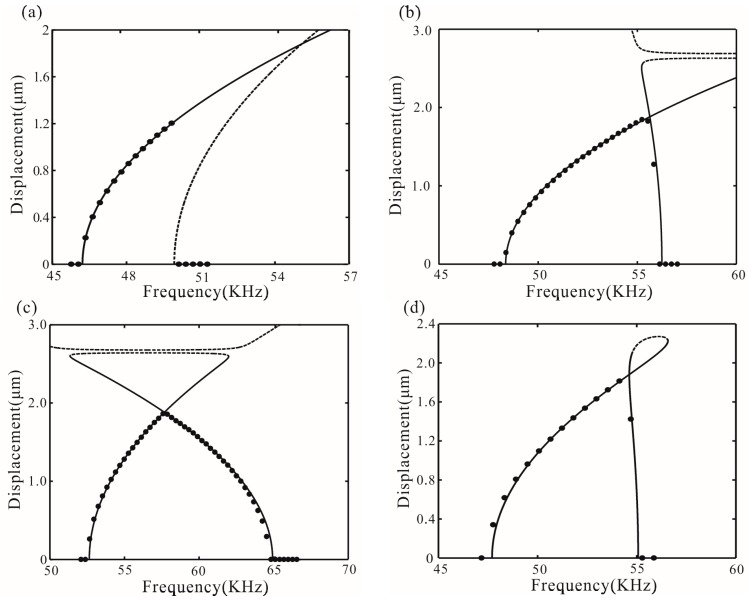
Frequency response curve (**a**) corresponding to *V* = 20 V and β = 0; (**b**) corresponding to *V* = 30 V and β = 0; (**c**) corresponding to *V* = 40 V and β = 0; and (**d**) corresponding to *V* = 30 V, ωτ*_n_* = π/4 and β = 0.225. Solid lines represent the analytical method and dotted lines represent the numerical method.

The above analysis is carried out based on the perturbation theory. This section gives the numerical results of the partial differential equation obtained by the finite difference method and long-time integration to verify the validity of the perturbation theory. Here, the foregoing analytical results based on the slow flow Equations (22) and (23) are compared with direct numerical integration. The numerical integration is completed by using fourth-order Runge–Kutta method with fixedstep. Figure 13 shows the frequency response curve. Here, we choose a small displacement disturbance as the initial value. The numerical method has yielded results that are in good agreement with the analytical solution, especially near the origin. 

## 7. Conclusions

This article theoretically investigates the nonlinear jumping phenomena and bifurcation conditions of a class of electrostatically-driven MEMS actuators with a time-delay feedback controller. A detailed analysis method is proposed to deal with the parametric excitation system consisting of a flexible beam and shuttle mass. It is found that our model can improve the resonance frequency and stability of the system. Meanwhile, an appropriate time-delay feedback force can make the branch from the Hopf bifurcation point stable under any driving voltage value.

Additionally, monostable vibration can eliminate dynamic bifurcation and improve system stability, which is desired in many MEMS applications. Theoretical expressions about the system parameter space and maximum amplitude of monostable vibration are deduced. It is found that the disappearance of the global bifurcation point means the emergence of monostable vibration. A method is proposed to translate the bistable state to a monostable state with time-delay feedback.

Finally, detailed numerical results confirm the analytical prediction. The analysis presented here describes a complete picture of this behavior, and provides designers of these devices with useful predictive tools.

## Figures and Tables

**Table 1:** Part of design parameters for a representative MEMS oscillator.

Parameter	Value	Units
Mass density ρ	2300	Kg/m^3^
Young’s modulus *E*	150	GPa
Beam length *L*	320	μm
Beam width *b*	10	μm
Beam thickness *h*	2	μm
Coefficients *r*_1_	5.3 × 10^−3^	μN·μm^−1^·V^−2^
Coefficients *r*_3_	−1.5 × 10^−3^	μN·μm^−3^·V^−2^
Damping *c*	5.42 × 10^−^^3^	Ns·m^−^^2^
Mass *M*	5.95 × 10^−10^	kg

## Appendix A

Through Equations (22) and (23), we obtain:

(A1) $$(2\lambda_{1}+a^{2}\lambda_{3})\sin2\psi =8\xi +4g\sin\omega \tau_{n}$$

(A2) $$(2\lambda_{1}+2a^{2}\lambda_{3})\cos2\psi =-3a^{2}k_{3}-4k_{1}+4\sigma +4g\cos\omega \tau_{n}$$

Then, the square of Equation (A1) is added to the square of Equation (A2) and, ignoring the high-order nonlinear term, we obtain:

(A3) $$\begin{array}{l} {\left[(2\lambda_{1}+a^{2}\lambda_{3})\sin2\psi \right]}^{2}+{\left[(2\lambda_{1}+2a^{2}\lambda_{3})\cos2\psi \right]}^{2}\\ =4\lambda_{1}^{2}+4\lambda_{1}\lambda_{3}{\left(\sin2\psi \right)}^{2}a^{2}+8\lambda_{1}\lambda_{3}{\left(\cos2\psi \right)}^{2}a^{2} \end{array}$$

From Equations (A1) and (A2), we obtain:

(A4) $$2\lambda_{1}\sin2\psi =8\xi +4g\sin\omega \tau_{n}-a^{2}\lambda_{3}\sin2\psi$$

(A5) $$2\lambda_{1}\cos2\psi =-3a^{2}k_{3}-4k_{1}+4\sigma +4g\cos\omega \tau_{n}-2a^{2}\lambda_{3}\cos2\psi$$

Substituting Equations (A4) and (A5) into Equation (A3), and ignoring the high-order nonlinear term, yields:

(A6) $$\begin{array}{l} f(\sigma ,a)=4\lambda_{1}^{2}-[{\left(8\xi +4g\sin\omega \tau_{n}\right)}^{2}+{\left(4\sigma +4g\cos\omega \tau_{n}-4k_{1}\right)}^{2}]\\ +[2\lambda_{3}\sin2\psi (8\xi +4g\sin\omega \tau_{n})+2(4\sigma +4g\cos\omega \tau_{n}-4k_{1})(2\lambda_{3}\cos2\psi +3k_{3})]a^{2}=0 \end{array}$$

Firstly, we consider the bifurcation point (σ,*a*) = (σ_1_,0) and obtain:

(A7) $$2\lambda_{1}\sin2\psi =8\xi +4g\sin\omega \tau_{n}$$

(A8) $$2\lambda_{1}\cos2\psi =-\sqrt{4{\lambda_{1}}^{2}-{\left(8\xi +4g\sin\omega \tau_{n}\right)}^{2}}$$

According to the condition of the Hopf bifrucation, substituting Equations (A7) and (A8) and Equation (30) into Equation (A6) and we can obtain the discriminant:

(A9) $$\Delta_{1}=-3k_{3}\sqrt{4\lambda_{1}^{2}-{\left(8\xi +4g\sin\omega \tau_{n}\right)}^{2}}+4\lambda_{1}\lambda_{3}-\frac{\lambda_{3}}{2\lambda_{1}}{\left(8\xi +4g\sin\omega \tau_{n}\right)}^{2}$$

If $\Delta_{1}$ < 0, there exists stable periodic vibration in the system when σ > σ_1_, which results in the supercritical Hopf bifurcation of σ_1_. On the contrary, if $\Delta_{1}$ > 0, there exists unstable periodic vibration in the system when σ < σ_1_, which results in the subcritical Hopf bifurcation of σ_1_.

Likewise, we can obtain the discriminant of σ_2_:

(A10) $$\Delta_{2}=3k_{3}\sqrt{4\lambda_{1}^{2}-{\left(8\xi +4g\sin\omega \tau_{n}\right)}^{2}}+4\lambda_{1}\lambda_{3}-\frac{\lambda_{3}}{2\lambda_{1}}{\left(8\xi +4g\sin\omega \tau_{n}\right)}^{2}$$

## Appendix B

In this article, we consider λ_1_ > 0 and λ_3_ < 0.

Firstly, Equation (33) can be written as a parabolic equation:

(B1) $$\begin{array}{c} \Delta_{1}=-3k_{3}\sqrt{4\lambda_{1}^{2}-{\left(8\xi +4g\sin\omega \tau_{n}\right)}^{2}}+4\lambda_{1}\lambda_{3}-\frac{\lambda_{3}}{2\lambda_{1}}{\left(8\xi +4g\sin\omega \tau_{n}\right)}^{2}\\ =\frac{\lambda_{3}}{2\lambda_{1}}[4\lambda_{1}^{2}-{\left(8\xi +4g\sin\omega \tau_{n}\right)}^{2}]-3k_{3}\sqrt{4\lambda_{1}^{2}-{\left(8\xi +4g\sin\omega \tau_{n}\right)}^{2}}+2\lambda_{1}\lambda_{3} \end{array}$$

When the discriminant of parabolic equation Δ = 9$k_{3}^{2}$−4$\lambda_{3}^{2}$< 0, $\Delta_{1}$ < 0. Supercritical Hopf bifurcation occurs.

Then, Δ = 9$k_{3}^{2}$−4$\lambda_{3}^{2}$ > 0 and *k*_3_ <0 are considered. If equivalent damping $2\xi +g\sin\omega \tau_{n}$ approaches to zero, we obtain:

$$\Delta_{1}\approx 4\lambda_{1}\lambda_{3}-6\lambda_{1}k_{3}=2\lambda_{1}(2\lambda_{3}-3k_{3})>0$$

Subcritical Hopf bifurcation occurs.

When $g\sin\omega \tau_{n}$ is close to $\lambda_{1}/2-2\xi$, $\Delta_{1}\approx 2\lambda_{1}\lambda_{3}<0$. Supercritical Hopf bifurcation occurs.

Likewise, $\Delta_{2}$ can be studied in this manner.
